# Deep Learning-Based Dynamic Time Division ISAC Beamforming for Vehicular Networks

**DOI:** 10.3390/s26092790

**Published:** 2026-04-30

**Authors:** Junseok Lim, Jaewoo So

**Affiliations:** Department of Electronic Engineering, Sogang University, Seoul 04107, Republic of Korea

**Keywords:** integrated sensing and communications (ISAC), vehicular communications, ISAC beamforming, dynamic time division, Cramér–Rao lower bound (CRLB), proximal policy optimization (PPO)

## Abstract

Integrated sensing and communications (ISAC) is a promising key technology for vehicular networks, because it allows roadside units to support both data transmission and radar-like sensing over the same spectrum and hardware platform. In conventional time division ISAC systems, each frame is divided into sensing and communication phases with a fixed ratio, which determines the tradeoff between the sensing accuracy and the communication throughput. However, in high-mobility vehicular environments, a fixed sensing–communication split is often suboptimal due to time-varying channel and intervehicle interference variations. In this paper, we propose a dynamic sensing–communication time division and ISAC beamforming scheme that minimizes the Cramér–Rao lower bound while satisfying the minimum effective communication sum rate. We further develop a deep reinforcement learning framework based on proximal policy optimization to find the optimal time division ratio and beamforming vectors. Simulation results show that the proposed dynamic time division beamforming scheme significantly outperforms the conventional fixed time division beamforming schemes in terms of sensing accuracy and the communication sum rate.

## 1. Introduction

Rapid advancements in intelligent transportation systems and autonomous driving technologies have led to a significant increase in wireless traffic between vehicles and networks, as well as between vehicles. Therefore, vehicle-to-everything (V2X) technologies are required to simultaneously provide high-data-rate services and accurate situational awareness within limited spectrum resources, even in highly dynamic wireless environments [1,2,3].

Recently, integrated sensing and communications (ISAC) technology has attracted significant attention as a promising solution that enables wireless communication and sensing in the same system. ISAC performs both radar-like sensing and data transmission over the same spectrum and hardware platform, thereby improving the spectral efficiency while reducing the hardware cost and system complexity [4,5,6]. Previous ISAC studies can be broadly categorized into those on time division ISAC (TD-ISAC), resource allocation-based ISAC, and beamforming-oriented ISAC.

Many studies have focused on TD-ISAC systems, which separate sensing and communication into different time intervals [7,8]. In particular, TD-ISAC has attracted considerable attention because it can address challenges such as full-duplex hardware design and interference suppression by separating the sensing and communication phases in the time domain [9,10]. For this reason, TD-ISAC is often regarded as a practical first step toward ISAC implementation. The authors of [7] proposed a TD-ISAC system for connected automated vehicles, in which they considered various sensing and communication time configurations and evaluated the performance under different sensing-to-communication-time ratios. The authors of [11] studied the time allocation problem in TD-ISAC systems and mathematically analyzed the relationship between radar detection accuracy and the achievable data rate for a given fixed time interval. The authors of [12] focused on the design of a cross-layer scheduling policy for a TD-ISAC system with a buffer-equipped base station in a single-input single-output (SISO) scenario, where they used a queuing model to derive the communication performance and assumed that the radar detection accuracy is determined by the allocated time resources. In a TD-ISAC system, the time ratio between sensing and communication determines the tradeoff between the two functions. In high-mobility vehicular environments, it becomes increasingly important to dynamically adjust the sensing-to-communication-time ratio in response to channel variations. In addition, the roadside unit (RSU) should determine the beamforming matrices for sensing and communication [13]. However, most previous TD-ISAC studies have considered a predetermined or enumerated sensing/communication time split and then evaluated performance under this fixed ratio. Although these studies provide useful insights into the sensing–communication tradeoff, they do not dynamically optimize the time split according to channel and mobility variations.

Some studies have addressed time, frequency, and power allocation in ISAC systems [14,15,16]. The authors of [17] analyzed the performance tradeoff between sensing and communication in frequency division ISAC networks, where separate frequency resources are assigned to sensing and data transmission. In particular, they mathematically derived the probability of detection for sensing and the probability of coverage for communication under a given transmit power and bandwidth budget. The authors of [18,19,20,21] investigated joint time–frequency resource allocation. Specifically, the authors of [18,19,20] proposed resource optimization designs that minimize the Cramér–Rao lower bound (CRLB) for delay and Doppler estimation, whereas the authors of [21] focused on suppressing delay Doppler sidelobes. The authors of [22] aimed to jointly optimize resource and power allocation for a monostatic orthogonal frequency division multiplexing (OFDM)–ISAC system while satisfying sensing signal-to-noise ratio (SNR) and communication sum rate requirements. However, although these studies dynamically allocate time–frequency resources, they do not consider beamforming matrices for sensing and data transmission.

ISAC beamforming studies have mainly focused on designing beams for simultaneous sensing and communication, where the beamformer matrix determines the tradeoff between sensing and communication performance [23,24]. A large portion of the ISAC beamforming literature considers simultaneous sensing–communication transmission, where optimization focuses on beam patterns, transmit covariance matrices, or communication precoders, rather than on dynamic frame-level time division. The authors of [24,25,26,27] investigated joint beam pattern design for ISAC, aiming either to improve the sensing accuracy by minimizing the CRLB or to enhance the communication capacity by maximizing the sum rate. In particular, the authors of [24] formulated an ISAC beamforming design for vehicle-mounted transmitters in the uplinks of vehicle-to-infrastructure (V2I) networks, while the authors of [25] proposed sensing-assisted predictive beam tracking in the downlinks of V2I networks. Recently, many researchers have utilized deep learning or reinforcement learning for beamforming design in ISAC networks [28,29]. The authors of [30,31] used deep reinforcement learning (DRL) for dynamic beamforming and power allocation in ISAC systems, and the authors of [32] proposed an energy-efficient DRL-based beamforming scheme for ISAC-enabled V2X networks. However, these ISAC beamforming studies do not formulate the joint TD-ISAC problem with both adaptive time partitioning and multi-user beamforming—that is, they do not dynamically optimize frame-level time division.

In this paper, we propose a dynamic time allocation and beamforming scheme for TD-ISAC systems. The proposed scheme jointly optimizes the sensing-to-communication-time allocation and transmit beamforming vectors for sensing and communication. Our paper differs from prior studies in that it integrates two strongly coupled decisions that are usually treated separately: how much frame time should be assigned to sensing vs. communication and how the corresponding sensing and communication beams should be designed under this time split. This coupling is central to our formulation. For example, a larger sensing ratio improves the effective sensing SNR and reduces the CRLB, but it shortens the communication phase and reduces the effective sum rate. At the same time, the beamforming quality affects both the sensing gain and communication SINR. Because these decisions interact nonlinearly and vary over time with vehicle positions and channels, it is insufficient to optimize them independently. The main contributions of this paper are as follows. First, we propose a joint optimization scheme for time allocation and beamforming vectors in ISAC-aided V2I networks. Specifically, the proposed scheme dynamically adjusts the sensing-to-communication-time ratio and optimizes the beamforming vectors for sensing and communication according to the channel condition. Second, we incorporate a CRLB-oriented sensing objective together with a minimum effective sum rate constraint, thereby explicitly balancing sensing accuracy and communication performance. Third, we develop a proximal policy optimization (PPO) algorithm to solve the optimization problem, where the reward is determined as the weighted sum of the sensing performance and the communication performance.

The rest of this paper is organized as follows. Section 2 describes the system and channel models. Section 3 presents the sensing and communication performance. Section 4 formulates the optimization problem and presents the proposed dynamic time division (DTD)–ISAC beamforming scheme, where the state, action, and reward of DRL are described in detail. Section 5 provides the simulation results, and Section 6 concludes the paper.

## 2. System Model

### 2.1. System Description

**Figure 1:**
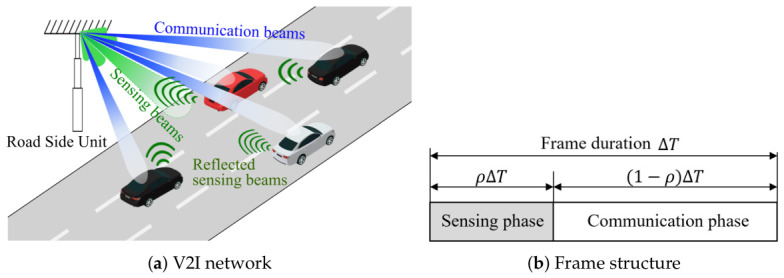
The system model.

We consider a downlink of an ISAC-enabled V2I network with a single RSU and *K* vehicles, as shown in Figure 1a. An RSU is equipped with a uniform linear transmit array of $N_{t}$ antennas and with a uniform linear receive array of $N_{r}$ antennas dedicated to monostatic radar sensing. The RSU serves *K* vehicles, where each vehicle is equipped with *M* receive antennas.

The RSU carries out the sensing and communication functions in a time division manner. As shown in Figure 1b, each frame is divided into a sensing phase with a ratio of ρ and a communication phase with a ratio of 1−ρ, where ρ∈[0,1]. During the sensing phase of ρΔT, the RSU transmits a dedicated sensing waveform and receives the reflected echoes to estimate the angle and range of the vehicles. During the communication phase of (1−ρ)ΔT, the RSU transmits independent *K* data streams to the *K* vehicles by using a multi-user transmit beamforming matrix.

### 2.2. Channel Model

The wireless channel between the RSU and the *k*-th vehicle is characterized by a line-of-sight (LoS) propagation model, suitable for highway and open-road scenarios [2,33]. Let $\beta_{k}\left(t\right)$ denote the large-scale path loss coefficient and $\theta_{k}\left(t\right)$ denote the geometric angle of departure (AoD) from the RSU to vehicle *k*. The downlink channel $H_{k}\left(t\right)\in C^{M\times N_{t}}$ from the RSU to vehicle *k* at time *t* can then be expressed as follows:

(1) $$\begin{array}{c} H_{k}\left(t\right)=\sqrt{\beta_{k}\left(t\right)}a_{\mathrm{rx},\mathrm{veh}}\left(\theta_{k}\left(t\right)\right)a_{\mathrm{tx}}^{H}\left(\theta_{k}\left(t\right)\right), \end{array}$$

where the vector $a_{\mathrm{tx}}\left(\cdot \right)$ represents the transmit array response at the RSU, and the vector $a_{\mathrm{rx},\mathrm{veh}}\left(\cdot \right)$ denotes the receive array response at the vehicle. Assuming uniform linear arrays (ULAs) with half-wavelength spacing, the transmit and receive array responses are, respectively, given by

(2) $$\begin{array}{ccc} a_{\mathrm{tx}}\left(\theta \right) & = & \frac{1}{\sqrt{N_{t}}}{\left[1,e^{j\pi \sin\theta },\ldots ,e^{j\pi (N_{t}-1)\sin\theta }\right]}^{T}, \end{array}$$

(3) $$\begin{array}{ccc} a_{\mathrm{rx},\mathrm{veh}}\left(\theta \right) & = & \frac{1}{\sqrt{M}}{\left[1,e^{j\pi \sin\theta },\ldots ,e^{j\pi (M-1)\sin\theta }\right]}^{T}. \end{array}$$

The path loss coefficient $\beta_{k}\left(t\right)$ follows a standard distance-dependent decay model as follows:

(4) $$\beta_{k}\left(t\right)=\beta_{0}{\left(\frac{r_{0}}{r_{k}\left(t\right)}\right)}^{\alpha },$$

where $r_{k}\left(t\right)$ is the distance between the RSU and vehicle *k* at time *t*, $r_{0}$ is a reference distance, $\beta_{0}$ is the path loss at the reference distance, and α is the path loss exponent.

## 3. Performance Evaluation in a TD-ISAC System

The RSU employs different transmit signals during the sensing and communication phases. The total transmit power of an RSU during a frame is constrained by a budget $P_{\mathrm{tot}}$, which is shared between the sensing and communication phases. In a TD-ISAC system, the frame duration ΔT is divided into a sensing phase and a communication phase. First, during ρΔT, an RSU carries out the sensing function. During this sensing phase, the RSU transmits a dedicated probing signal and receives echoes on its sensing array. Then, during the remaining (1−ρ)ΔT, the RSU carries out the communication function. During this communication phase, the RSU serves *K* vehicles simultaneously using downlink multi-user beamforming.

### 3.1. Sensing Performance

During the sensing phase, the RSU transmits a dedicated sensing beam $f_{\mathrm{sens}}\in C^{N_{t}\times 1}$ using a probing waveform $x_{\mathrm{sens}}\left(t\right)$. The RSU then receives the reflected echoes with a sensing array consisting of $N_{r}$ elements. After matched filtering and coherent integration over τ independent echoes within the sensing period ρΔT, the RSU forms sufficient statistics for estimating the angle $\theta_{k}$ and range $r_{k}$ of each vehicle. The quality of these estimates is captured through the CRLB [34,35], which provides a lower bound on the variance of any unbiased estimator of a parameter.

The effective sensing SNR for target vehicle *k* after the coherent integration of τ echoes within the sensing period ρΔT is proportional to the sensing time, the path loss, and the array gain in the transmit direction, as follows [36,37]:

(5) $${\mathrm{SNR}}_{\mathrm{sens},k}\propto \rho \cdot \frac{\tau \;\beta_{k}\;|a_{\mathrm{tx}}^{H}\left(\theta_{k}\right)f_{\mathrm{sens}}{|}^{2}}{\sigma_{\mathrm{sens}}^{2}},$$

where ρ is the sensing-to-communication-time ratio, $\beta_{k}$ is the path loss coefficient for vehicle *k*, $a_{\mathrm{tx}}\left(\cdot \right)$ is the transmit array response at the RSU, $f_{\mathrm{sens}}$ is the sensing beamforming vector, and $\sigma_{\mathrm{sens}}^{2}$ is the noise power.

The CRLB for angle estimation is inversely proportional to the sensing SNR and the square of the effective aperture $D_{\mathrm{ap}}$. Hence, the CRLB for the angle estimation of vehicle *k* can then be expressed as follows:

(6) $${\mathrm{CRLB}}_{\theta ,k}\propto \frac{1}{{\mathrm{SNR}}_{\mathrm{sens},k}\;D_{\mathrm{ap}}^{2}}.$$

Similarly, the CRLB for range estimation is inversely proportional to the sensing SNR and the square of the system bandwidth *B*; therefore, the CRLB for the range estimation of vehicle *k* can be expressed as follows:

(7) $${\mathrm{CRLB}}_{r,k}\propto \frac{1}{{\mathrm{SNR}}_{\mathrm{sens},k}\;B^{2}}.$$

Consequently, the integrated CRLB for the sensing performance can be expressed as a weighted sum of the per-user angle and range CRLBs as follows:

(8) $$\begin{array}{c} \Omega_{\mathrm{sens}}=w_{\theta }\frac{1}{K}\sum_{k=1}^{K}{\mathrm{CRLB}}_{\theta ,k}+\left(1-w_{\theta }\right)\frac{1}{K}\sum_{k=1}^{K}{\mathrm{CRLB}}_{r,k}, \end{array}$$

where ${\mathrm{CRLB}}_{\theta ,k}$ and ${\mathrm{CRLB}}_{r,k}$ are, respectively, the angle and range CRLBs of vehicle *k*, and $w_{\theta }\geq 0$ is a system parameter that controls the relative weighting of the angle and range estimations.

In particular, increasing the sensing duration ρΔT allows the receiver to accumulate more energy from the reflected signals, thereby linearly increasing the effective sensing SNR. Because the CRLB is inversely proportional to the SNR from (6) and (7), allocating more time resources to sensing directly suppresses the estimation error variance.

### 3.2. Communication Performance

During the communication phase, the RSU transmits data symbols to *K* vehicles using a transmit beamforming matrix

(9) $$F_{\mathrm{comm}}=\left[f_{1},\ldots ,f_{K}\right]\in C^{N_{t}\times K},$$

where $f_{k}\in C^{N_{t}\times 1}$ is the beamforming vector for vehicle *k*. Let $s_{k}\in C$ denote the information symbol intended for vehicle *k*, with unit average power $E[|s_{k}{|}^{2}]=1$. The transmitted signal vector at the RSU is

(10) $$x_{\mathrm{comm}}=\sum_{k=1}^{K}f_{k}s_{k}=F_{\mathrm{comm}}s,$$

where $s={\left[s_{1},\ldots ,s_{K}\right]}^{T}$.

The received signal vector at vehicle *k* is

(11) $$y_{k}=H_{k}x_{\mathrm{comm}}+n_{k},$$

where $n_{k}∼\mathrm{CN}\left(0,\sigma_{\mathrm{comm}}^{2}I_{M}\right)$ is additive white Gaussian noise (AWGN). Vehicle *k* applies a linear receive combiner $w_{k}\in C^{M\times 1}$ to obtain the decision variable

(12) $${\hat{s}}_{k}=w_{k}^{H}y_{k}.$$

Here, we choose common maximum ratio combining (MRC) for $w_{k}$ as follows:

(13) $$w_{k}=\frac{H_{k}f_{k}}{∥H_{k}f_{k}∥},$$

which maximizes the instantaneous receive SNR in single-user settings.

The instantaneous signal-to-interference-plus-noise ratio (SINR) at vehicle *k* under linear receive combining is given by

(14) $${\mathrm{SINR}}_{k}=\frac{{\left|w_{k}^{H}H_{k}f_{k}\right|}^{2}}{\sum_{j\neq k}{\left|w_{k}^{H}H_{k}f_{j}\right|}^{2}+\sigma_{\mathrm{comm}}^{2}{∥w_{k}∥}^{2}}.$$

Assuming Gaussian signaling and ideal coding, the effective sum rate per frame is given by

(15) $$R_{\mathrm{comm}}=\left(1-\rho \right)\cdot \sum_{k=1}^{K}\log_{2}\left(1+{\mathrm{SINR}}_{k}\right).$$

## 4. Proposed DTD-ISAC Beamforming Scheme

### 4.1. Problem Formulation

Our objective is to minimize the integrated CRLB of $\Omega_{\mathrm{sens}}\left(t\right)$ at each decision epoch *t*, while satisfying the effective sum rate requirement for communication greater than or equal to a threshold of $R_{\min}$. In the proposed DTD-ISAC system, the sensing–communication time split can be dynamically adjusted according to the time-varying channel. Hence, the parameters to be dynamically determined at time *t* are as follows: the sensing-to-communication-time ratio of $\rho_{t}$, the sensing beamforming vector of $f_{\mathrm{sens},t}$, and the communication beamforming vectors of ${\left\{f_{k,t}\right\}}_{k=1}^{K}$. The optimization problem can then be expressed as follows:

(16) $$\begin{array}{cc} \min_{\rho_{t},f_{\mathrm{sens},t},f_{1,t},\cdots ,f_{K,t}} & \underset{T\to \infty }{lim sup}\frac{1}{T}\;E\left[\;\sum_{t=0}^{T-1}\Omega_{\mathrm{sens}}\left(t\right)\;\right]\\ s.t.\; & R_{\mathrm{comm}}\left(t\right)\geq R_{\min},\\ & \rho_{\min}\leq \rho_{t}\leq \rho_{\max},\\ & \sum_{k=1}^{K}∥f_{k,t}{∥}^{2}+{∥f_{\mathrm{sens},t}∥}^{2}\leq P_{\mathrm{tot}}. \end{array}$$

It is difficult to directly solve (16) due to the fact that $\Omega_{\mathrm{sens}}\left(t\right)$ and $R_{\mathrm{comm}}\left(t\right)$ have a non-convex dependency on the beamforming vectors, the temporal coupling induced by the channel and mobility dynamics, and the requirement for real-time decision-making. Consequently, we reformulate the problem of (16) using a DRL framework based on the PPO algorithm.

### 4.2. MDP Modeling

Let π denote a control policy that maps observations $o_{t}$ at decision epoch *t* to a continuous action vector $a_{t}$:

(17) $$a_{t}=\pi \left(o_{t}\right).$$

To represent the tradeoff between sensing and communication performance as a single scalar measure, an integrated reward is defined as follows:

(18) $$r_{t}=-\Omega_{\mathrm{sens}}\left(t\right)-\lambda_{\mathrm{rate}}{\left[R_{\min}-R_{\mathrm{comm}}\left(t\right)\right]}^{+}+\lambda_{\exp}\left(1-2\right|\rho_{t}-0.5\left|\right),$$

where ${\left[x\right]}^{+}=\max\left(x,0\right)$, $\lambda_{\mathrm{rate}}$ is a penalty weight that controls the severity of violations of the communication rate requirement, and $\lambda_{\exp}$ is a weight for an exploration bonus. In the reward of (18), each term is designed to reflect a specific control objective. The first term directly encourages the reduction of the integrated sensing error represented by the CRLB. The second term imposes a penalty when the communication sum rate falls below the minimum required threshold; therefore, $\lambda_{\mathrm{rate}}$ is introduced to enforce the communication rate constraint with sufficient importance during training. The third term provides an exploration incentive for the sensing time ratio $\rho_{t}$ so that the learned policy does not become prematurely biased toward extreme boundary values in the early stage of learning. Accordingly, $\lambda_{\exp}$ is used as a regularization-type parameter to support stable exploration rather than as a dominant performance term. These weighting parameters can be selected empirically so that the reward components have a comparable influence during training and the agent can learn a balanced policy without excessive bias toward only one objective. More specifically, $\lambda_{\mathrm{rate}}$ is chosen to be large enough to strongly discourage rate constraint violation, while $\lambda_{\exp}$ is set to be relatively small so that it assists exploration without overriding the main sensing–communication tradeoff.

Given a policy π, the long-term performance over a horizon of $T_{\mathrm{epi}}$ frames is characterized by the expected discounted return

(19) $$J\left(\pi \right)=E_{\pi }\left[\sum_{t=0}^{T_{\mathrm{epi}}-1}\gamma r_{t}\right],$$

where γ∈(0,1] is a discount factor. The control objective within the DRL framework is to find a policy $\pi^{⋆}$ that maximizes the expected return, as follows:

(20) $$\pi^{⋆}=\arg\max_{\pi }J\left(\pi \right).$$

The DTD-ISAC control problem can be modeled as a Markov decision process (MDP) described by the tuple (S,A,P,R,γ), where S is the state, A is the action, *P* is the transition, *R* is the reward, and γ is the discount factor.

#### 4.2.1. State and Observation

The underlying system state at time *t*, denoted by $s_{t}\in S$, includes the positions, velocities, and channel realizations of all vehicles, as follows:

(21) $$s_{t}=\left(p_{t},v_{t},{\left\{H_{k}\left(t\right)\right\}}_{k=1}^{K}\right),$$

where $p_{t}$ is the positions of vehicles, $v_{t}$ is the velocities of vehicles, and $H_{k}\left(t\right)$ is the channel of vehicle *k*. The policy operates on an observation vector $o_{t}=O\left(s_{t}\right)$, where O(·) denotes the observation function. Note that performance metrics are normalized using statistics collected from an initial calibration phase to ensure stable training.

#### 4.2.2. Action Space

The RSU controls the high-dimensional beamforming vectors, but learning these vectors directly is computationally complex. Hence, we employ a parameterized action space, where the agent controls the beamforming direction and power allocation. The action vector $a_{t}\in R^{2+2K}$ at time *t* is defined as

(22) $$a_{t}=\left(\rho_{t},p_{\mathrm{sens},t},\Delta \phi_{1,t},\ldots ,\Delta \phi_{K,t},p_{1,t},\ldots ,p_{K,t}\right),$$

where $\rho_{t}\in \left(0,1\right)$ is the sensing-to-communication-time ratio, $p_{\mathrm{sens},t}\in \left(0,1\right)$ is the proportion of the total power allocated to sensing, and $p_{k,t}$ is a power allocation coefficient that determines the power of the communication beam for vehicle *k*. Additionally, $\Delta \phi_{k,t}$ is an angular offset applied to the estimated line-of-sight (LoS) angle for vehicle *k*. The communication beam $f_{k,t}$ is constructed as a steering vector pointing toward ${\hat{\theta }}_{k}+\Delta \phi_{k,t}$.

This structured action design is adopted to maintain the tractability of the DRL problem in a high-mobility vehicular environment. Directly optimizing full complex-valued sensing and communication beamforming vectors would lead to a much higher-dimensional continuous action space and significantly increase the training difficulty. Therefore, the proposed parameterization captures the dominant beam steering and power allocation decisions relevant to directional vehicular channels while keeping the learning problem manageable. Although this action space does not represent the most general beamforming structure and may not achieve globally optimal unconstrained precoding, it provides a practical and stable formulation for jointly optimizing time division and beam control in TD-ISAC vehicular networks.

#### 4.2.3. Transition and Reward

The state evolves according to a stochastic transition probability $P(s_{t+1}|s_{t},a_{t})$ that reflects the mobility and channel dynamics of vehicles. The transition model is realized through a simulation environment that generates vehicle trajectories and channel realizations in a vehicular network.

The reward at time *t* is given by (18). The reward combines the sensing performance $\Omega_{\mathrm{sens}}\left(t\right)$ and the communication performance $R_{\mathrm{comm}}\left(t\right)$, which encourages the DRL agent to reduce the CRLB-based sensing error while meeting the minimum communication rate requirement.

### 4.3. PPO-Based Learning

We apply an actor–critic architecture via PPO to learn a stochastic policy $\pi_{\theta }\left(a|o\right)$, parameterized by θ, where *a* is the action and *o* is the observation. PPO is a policy gradient method that employs a clipped surrogate objective to stabilize policy updates [38].

The policy network takes the observation $o_{t}$ as input and outputs the parameters (for example, mean and variance) of a multivariate Gaussian distribution over the continuous action space. The value network has a similar structure but outputs a scalar estimate $V_{\phi }\left(o_{t}\right)$ of the state value, with parameters ϕ. In this paper, both networks are implemented using two fully connected hidden layers with 128 units each and nonlinear activation functions.

Let $\theta_{\mathrm{old}}$ denote the policy parameters before an update. For a batch of trajectories, the PPO objective is given by

(23) $$L_{\mathrm{PPO}}\left(\theta \right)=E_{t}\left[\min\left(p_{t}\left(\theta \right){\hat{A}}_{t},\mathrm{clip}\left(p_{t}\left(\theta \right),1-\epsilon ,1+\epsilon \right){\hat{A}}_{t}\right)\right],$$

where ϵ>0 is a clipping parameter, ${\hat{A}}_{t}$ is an estimate of the advantage function at time step *t*, clip(x,a,b) is a clip function of [38], and $p_{t}\left(\theta \right)$ is the probability ratio as follows:

(24) $$p_{t}\left(\theta \right)=\frac{\pi_{\theta }\left(a_{t}|o_{t}\right)}{\pi_{\theta_{\mathrm{old}}}\left(a_{t}|o_{t}\right)}.$$

The value network parameters ϕ are updated by minimizing the squared value loss as follows:

(25) $$L_{V}\left(\phi \right)=E_{t}\left[{\left(V_{\phi }\left(o_{t}\right)-{\hat{V}}_{t}\right)}^{2}\right],$$

where ${\hat{V}}_{t}$ is a target value, such as the empirical return.

In the PPO framework, the training process alternates between collecting trajectories using the current policy and updating the policy and value networks based on the collected data. At time step *t*, the agent first takes an action $a_{t}$ based on the current observation $o_{t}$ using the policy π. Then, the agent calculates the reward $r_{t}$ of (18) and the next observation $o_{t+1}$. The transition $\left(o_{t},a_{t},r_{t}\right)$ is stored for later processing. After computing the advantage estimates $\left\{{\hat{A}}_{t}\right\}$, the parameters θ are updated using the mini-batch stochastic gradient descent (SGD) method by increasing the PPO objective of (23), and the parameters ϕ are updated by decreasing the value loss of (25). This procedure is repeated until the policy converges or a termination criterion, such as a maximum number of iterations, is reached. The details of the PPO-based DTD-ISAC beamforming scheme are summarized in Algorithm 1.
**Algorithm 1** PPO-based DTD-ISAC beamforming 1:Initialize the policy parameters θ and value parameters ϕ. 2:**for** $\mathrm{episode}=0,1,\cdots ,N_{\mathrm{ep}}-1$ **do** 3:   Reset the simulation environment and obtain the initial observation $o_{0}$. 4:   **for** time step $t=0,1,\cdots ,N_{T}-1$ **do** 5:      Take action $a_{t}∼\pi_{\theta }\left(\cdot |o_{t}\right)$. 6:      Observe reward $r_{t}$ according to (18). 7:      Observe the next observation $o_{t+1}$. 8:      Store $\left(o_{t},a_{t},r_{t}\right)$ for later processing. 9:   Compute empirical returns and advantage estimates $\left\{{\hat{A}}_{t}\right\}$.10:  Update θ by increasing the PPO objective of (23) and update ϕ by decreasing the value loss of (25).11:  **end for**12:**end for**

## 5. Simulation Results

We evaluate the performance of the proposed DTD-ISAC beamforming scheme in a V2I downlink scenario with a single RSU and K=3 vehicles, where the vehicles move at a constant speed of v∈ [10 m/s, 12 m/s] on a road. The vehicles are randomly dropped onto different lanes. The simulation parameters are summarized in Table 1. We run $N_{\mathrm{ep}}=100$ episodes with independent initial positions and velocities, where each episode consists of $N_{T}=100$ time steps with duration ΔT=0.1s; therefore, the episode duration is $T_{\mathrm{ep}}=N_{T}\Delta T=10\;s$.

The parameters for the PPO algorithm are as follows: the mini-batch size is 256, the discount factor is 0.99, the generalized advantage estimator (GAE) parameter is 0.95, the clipping range is 0.2, the number of hidden layers for the actor and critic networks is 2, the rollout length per update is 100 frames, the policy learning rate is 0.0003, the value learning rate is 0.001, the value loss coefficient is 0.5, the entropy regularization weight is 0.01, the number of PPO epochs per update is 10, and the number of training episodes is 100 per scenario.

We compare the performance of the proposed DTD-ISAC beamforming scheme with that of the conventional fixed time division (FTD)–ISAC beamforming scheme, which has a deterministically fixed sensing-to-communication-time ratio, ρ∈{0.1,0.3,0.5,0.7,0.9}. The performance of the ISAC beamforming schemes is evaluated in terms of sensing and communication performance metrics such as the average CRLB for angle estimation, the average CRLB for range estimation, and the average sum rate, as follows:

(26) $$\begin{array}{ccc} {\bar{R}}_{\mathrm{comm}} & = & \frac{1}{N_{\mathrm{ep}}N_{T}}\sum_{e=1}^{N_{\mathrm{ep}}}\sum_{t=1}^{N_{T}}R_{\mathrm{comm}}^{\left(e\right)}\left(t\right), \end{array}$$

(27) $$\begin{array}{ccc} {\bar{\mathrm{CRLB}}}_{\theta } & = & \frac{1}{N_{\mathrm{ep}}N_{T}K}\sum_{e=1}^{N_{\mathrm{ep}}}\sum_{t=1}^{N_{T}}\sum_{k=1}^{K}{\mathrm{CRLB}}_{\theta ,k}^{\left(e\right)}\left(t\right), \end{array}$$

(28) $$\begin{array}{ccc} {\bar{\mathrm{CRLB}}}_{r} & = & \frac{1}{N_{\mathrm{ep}}N_{T}K}\sum_{e=1}^{N_{\mathrm{ep}}}\sum_{t=1}^{N_{T}}\sum_{k=1}^{K}{\mathrm{CRLB}}_{r,k}^{\left(e\right)}\left(t\right). \end{array}$$

**Figure 2:**
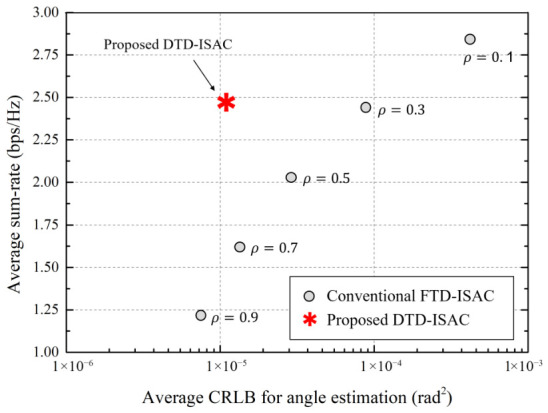
Average CRLB for angle estimation vs. average sum rate.

**Figure 3:**
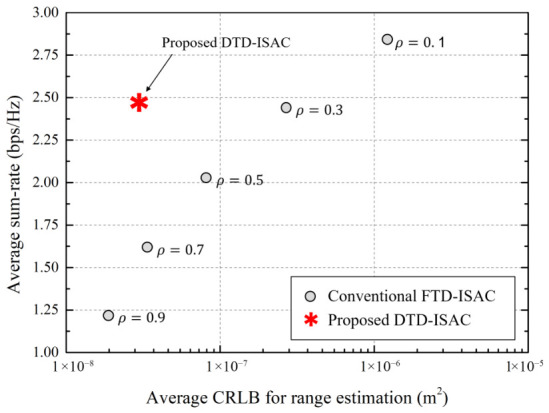
Average CRLB for range estimation vs. average sum rate.

Figure 2 shows the average CRLB for angle estimation, and Figure 3 shows the average CRLB for range estimation, where the x-axis in both figures represents the average sum rate. In conventional FTD-ISAC beamforming, as the value of ρ decreases, the communication phase lengthens, increasing the average sum rate, but the sensing phase shortens, degrading the sensing performance, namely ${\bar{\mathrm{CRLB}}}_{\theta }$ and ${\bar{\mathrm{CRLB}}}_{r}$. Additionally, conventional FTD-ISAC beamforming satisfies the communication sum rate requirement only when ρ is less than or equal to 0.3. However, the proposed DTD-ISAC beamforming scheme dynamically adjusts the sensing-to-communication-time ratio, ρ, depending on the channel environment. Hence, the proposed DTD-ISAC beamforming scheme shows good sensing accuracy while satisfying the minimum communication sum rate, $R_{\min}$. Because the conventional FTD-ISAC beamforming scheme cannot account for changes in wireless channels by fixing the sensing-to-communication-time ratio, the conventional scheme either significantly degrades the sensing accuracy when satisfying the communication data rate requirement or, conversely, significantly degrades the communication data rate when increasing the sensing accuracy. In particular, when ρ is 0.3 in the conventional scheme, the proposed scheme has similar sum rate performance but improves the CRLB by more than 80%. When ρ is 0.7 in the conventional scheme, the proposed scheme has similar CRLB performance but improves the sum rate by more than 45%.

**Figure 4:**
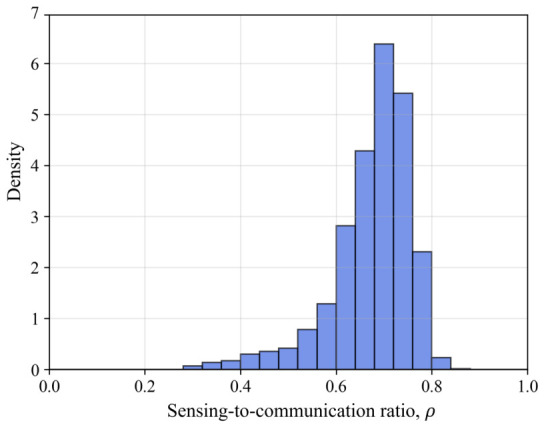
Distribution of ρ.

Figure 4 shows the experimental distribution of ρ during the simulation. It should be noted that the distribution of ρ varies depending on the channel environment. As shown in Figure 4, the value of ρ is not constant but varies as the vehicle moves. In this simulation, the average value of ρ is about 0.66.

## 6. Conclusions

In this paper, we propose a PPO-based DTD-ISAC beamforming scheme for V2I networks. The proposed scheme dynamically allocates time resources between sensing and data transmission while jointly optimizing the beamforming vectors in order to improve the sensing accuracy under the minimum communication data rate requirement. We developed an optimization problem that determines the sensing-to-communication-time ratio, the sensing beamforming vector, and the communication beamforming vector. However, because it is too difficult to solve the problem directly, we used the PPO algorithm by modeling the integrated reward, which includes the tradeoff between sensing and communication performance, as a single scalar measure. The proposed DRL agent observes the mobility-related states of the vehicles and link conditions, and it outputs in real time both the sensing-to-communication-time ratio and beamforming vectors. In the proposed DTD-ISAC system, the agent effectively increases the sensing time when the vehicle is far away or moving rapidly but reduces the sensing time when the communication data rate decreases to the minimum required rate. The proposed PPO-based DTD-ISAC beamforming scheme shows significantly better performance than the conventional FTD-ISAC beamforming scheme in terms of the sensing accuracy and data rate.

In this work, although the simulation was conducted with a limited number of vehicles, the overall performance trends of the proposed method would remain similar across scenarios, since the main advantage of the proposed DTD-ISAC framework arises from dynamically adjusting the sensing-to-communication-time ratio and beamforming decisions according to the channel and mobility conditions. When there are *K* vehicles, the action dimension is 2+2K, since the agent determines one sensing-to-communication-time ratio, one sensing power ratio, *K* steering offsets, and *K* communication power allocation variables. Accordingly, as *K* increases, the learning space becomes larger and the computational burden for both training and action selection also increases. Nevertheless, because the proposed action structure scales linearly with *K*, the framework remains applicable to larger vehicular scenarios, although with increased training time and implementation complexity.

Future research may include performance evaluations under more realistic channel models. Although this work adopts an LoS-dominant channel model, LoS-only channel models cannot fully reflect the various propagation characteristics encountered in practical vehicular environments, where NLoS components, blockage events, and time-varying fading can significantly affect both the sensing and communication performance. Hence, extending the proposed framework to more realistic propagation environments that include NLoS propagation, blockages, Doppler spread, and time-varying fading is an important direction for future research [39]. Moreover, the research would be strengthened by including comparisons with other dynamic or learning-based approaches, such as optimization-based adaptive schemes or alternative DRL algorithms.

## Figures and Tables

**Table 1:** Simulation parameters.

Parameter	Symbol	Value
Number of transmit antennas at RSU	$N_{t}$	8
Number of receive antennas at RSU	$N_{r}$	4
Number of receive antennas at vehicle	*M*	4
Number of vehicles	*K*	3
Carrier frequency	$f_{c}$	28GHz
Episode duration	$T_{\mathrm{ep}}$	10s
Frame duration	ΔT	0.1s
Total transmit power of RSU	$P_{\mathrm{tot}}$	1.0W
Communication noise power	$\sigma_{\mathrm{comm}}^{2}$	$10^{-10}\;W$
Path loss exponent	α	2.5
Minimum sum rate requirement	$R_{\min}$	2.4 bps/Hz

## Data Availability

Data are contained within the article.
